# Bacterial Osteomyelitis in the Lower Extremities: Analysis of Histology and MRI Findings in a Case-Control Pilot Study

**DOI:** 10.3390/jcm14144877

**Published:** 2025-07-09

**Authors:** Roslind K. Hackenberg, Fabio Schmitt-Sánchez, Christoph Endler, Verena Tischler, Jayagopi Surendar, Koroush Kabir, Kristian Welle, Christof Burger, Dieter C. Wirtz, Frank A. Schildberg

**Affiliations:** 1 Department of Hand, Plastic and Reconstructive Surgery, Burn Center, BG Trauma Center Ludwigshafen, University of Heidelberg, 67071 Ludwigshafen, Germany; 2 Department of Orthopedics and Trauma Surgery, University Hospital Bonn, 53127 Bonn, Germany; 3 Department of Diagnostic and Interventional Radiology, University Hospital Bonn, 53127 Bonn, Germany; 4 Institute of Pathology, University Hospital Bonn, 53127 Bonn, Germany

**Keywords:** osteomyelitis, lower extremities, histology findings, MRI findings, diagnostics, fracture-related infections

## Abstract

**Background:** Osteomyelitis, particularly affecting the lower extremities, is a serious and increasingly common complication. Accurate diagnosis is essential for successful treatment, yet standardized evidence-based protocols are lacking and diagnostic knowledge remains limited. This study aimed to identify characteristic histological and MRI findings in osteomyelitis to support diagnostic accuracy and guide treatment decisions. **Methods:** In a prospective case-control pilot study conducted from February 2020 to January 2021, all patients with suspected osteomyelitis of the lower limbs were included. Each underwent contrast-enhanced MRI and sampling for microbiological and histological analysis. Findings from five confirmed osteomyelitis cases were compared to five controls where osteomyelitis was ruled out. **Results:** All osteomyelitis cases showed typical MRI signs, including contrast-enhancing bone edema. Two had early, and two had pronounced intramedullary abscesses. In three controls, contrast-enhancing edema was limited to soft tissue; two showed mild adjacent bone edema. Histologically, all osteomyelitis samples revealed bone fragmentation and inflammatory cell infiltration—absent in controls. Additionally, four showed medullary fibrosis and one fibrin deposits. **Conclusions:** A comprehensive understanding of both histological and radiological findings is key to effective osteomyelitis treatment. This pilot study is the first to systematically compare MRI and histology findings side by side, offering valuable insights that may enhance diagnostic precision and support evidence-based treatment decisions.

## 1. Introduction

Bacterial osteomyelitis is an infection of the bone that arises either through hematogenous dissemination or by direct spread from adjacent infected tissues (per continuitatem). Healthy bone has high integrity and is naturally resistant to microbial invasion. As early as 1970, Waldvogel et al. demonstrated that a high bacterial load is required to colonize bone [1]. Typically, prior damage—such as hematoma or necrosis from trauma or surgery—is needed to allow infection.

Once bacteria reach the bone, they deploy multiple survival strategies to evade host defenses. These include expression of adhesion molecules to bind host matrix proteins, production of toxins for tissue invasion, formation of protective biofilms, intracellular survival within host cells, and encapsulated abscess-like colonies [2,3].

On the host side, acute infection triggers proinflammatory cascades involving cytokines like TNFα and IL-1β, along with neutrophil extracellular traps (NETs) to contain the infection [3,4]. In chronic stages, macrophages dominate, promoting fibrosis and abscess formation [3,5]. The adaptive immune system is also activated via T and B cells. Osteoblasts and osteoclasts respond to infection by attempting to remodel bone, while osteocytes help recruit T cells and amplify inflammation.

If pathogens are not eliminated, chronic osteomyelitis can develop, with abscess formation, periosteal reaction, bone necrosis (sequestra), fragmentation, and sinus tract formation.

Open fractures are the most common cause of bacterial osteomyelitis; however, any bone or periosteal surgical procedure can serve as a trigger. Additionally, minor soft tissue injuries are increasingly recognized as contributing factors to the development of osteomyelitis [2,6]. Extremities are affected in 91.5% of the cases, with the lower extremities accounting for 73.8% and the upper extremities for 17.7% [7]. Despite advancements in therapeutic strategies, the incidence of osteomyelitis continues to rise [8,9,10]. A study from Germany reported an incidence rate of 16.7 per 100,000 inhabitants for osteomyelitis in 2018, reflecting a 10.44% increase over a decade [7].

Advances in medical treatment, novel antibiotics, and increasingly sophisticated reconstructive surgical techniques have significantly improved limb salvage rates in osteomyelitis patients. However, limb salvage typically necessitates multiple complex orthoplastic procedures and prolonged treatment courses. In cases where limb salvage is not feasible or fails, amputation often remains the only therapeutic alternative. Local wound care and extended suppressive antibiotic therapy are considered palliative approaches in patients where neither limb preservation nor amputation is pursued.

Osteomyelitis can present in acute or chronic forms, with the potential for any acute case to become chronic and persist silently over years. Conversely, chronic osteomyelitis can undergo reactivation, leading to an “acute-on-chronic” presentation. Chronic osteomyelitis accounts for the highest proportion of cases at 47.6% [7]. Due to the high recurrence rate, the term “eradication” is no longer used in therapeutic discussions; instead, the goal is defined as achieving “remission,” similar to oncological disease management [11].

The foundation of successful treatment is comprehensive and accurate diagnostics. An incomplete or erroneous diagnostic approach carries a significant risk of mismanagement and therapeutic failure. The lack of standardized guidelines and evidence-based treatment protocols for osteomyelitis results in a heterogeneous approach to both diagnosis and therapy, often relying on expert consensus rather than evidence-based medicine.

In addition to standard laboratory markers of inflammation—which often lack specificity in osteomyelitis and may even remain within normal ranges [12]—conventional radiography remains the first-line imaging modality. Computed tomography (CT) with contrast medium is frequently used as an adjunct due to its rapid availability; however, it is only sensitive in advanced disease stages, where bone destruction, osteolysis, or sequestration are evident. Early osteomyelitis often remains undetectable on CT imaging.

Although magnetic resonance imaging (MRI) is considered the imaging modality of choice for osteomyelitis [13,14], it is not consistently performed in clinical practice. MRI has the unique capability to detect early-stage osteomyelitis that is not yet visible on X-ray or CT scans. However, due to the lack of routine implementation and the limited availability of musculoskeletal radiologists, essential imaging sequences may be omitted, potentially compromising diagnostic accuracy. Additionally, misconceptions regarding the optimal timing or contraindications—such as the presence of orthopedic implants—persist in clinical practice [15]. When performed appropriately, MRI achieves a sensitivity of 82–100%, significantly outperforming CT, which has a sensitivity of only 47% [16].

Thus, the gold standard for diagnosing bone and joint infections remains microbiological sampling. Despite being the definitive diagnostic tool, microbiological testing has limitations: the pathogen identification rate is approximately 90% in acute bone infections but drops to 51.4% in chronic cases [17]. Histopathological examination of bone biopsy specimens represents an additional diagnostic approach. While not routinely performed, histopathology achieves a diagnostic accuracy of 90.4–100% in osteomyelitis detection [12,18].

Given the crucial role of MRI and histology in diagnosing and managing osteomyelitis—and the persistent gaps in awareness regarding their interpretation—this study aims to systematically delineate and differentiate the characteristic findings of bacterial osteomyelitis in the lower extremities using MRI and histological analysis in a prospective case-control pilot study.

## 2. Materials and Methods

### 2.1. Study Design

At a tertiary care hospital, a prospective single-center pilot study was conducted between February 2020 and January 2021. All patients presenting with suspected post-traumatic osteomyelitis of the lower extremities based on clinical history and examination were enrolled. Additional inclusion criteria were a minimum age of 18 years and a history of previous soft tissue and/or osseous injury.

### 2.2. Patient Population and Data Collection

All enrolled patients underwent contrast-enhanced MRI and, as part of surgical débridements, microbiological and histopathological bone and soft tissue samples were obtained. Patients were followed up for one year. A definitive diagnosis of osteomyelitis was established if MRI findings were consistent with osteomyelitis, microbiological cultures from bone specimens yielded pathogen growth, and histopathological examination confirmed bone inflammation.

Osteomyelitis was ruled out if MRI showed no distinct signs of osteomyelitis beyond those attributable to post-traumatic changes, no microorganism was isolated from bone cultures, histopathology showed no inflammation, and no osteomyelitis developed during the one-year follow-up period. Patients meeting these exclusion criteria were assigned to the control group. Patients were excluded from the study if they developed osteomyelitis within one year, despite initially negative findings, or up to three consecutive débridements failed to identify a microbiological pathogen, combined with incongruent MRI and histopathological results.

In addition to MRI and histopathological findings, the following clinical parameters were documented for all enrolled patients: age and sex, osteomyelitis localization, previous trauma, comorbidities, and microbiological results.

### 2.3. Diagnostic Procedures

MRI assessments were performed by radiologists specialized in musculoskeletal imaging, while histopathological evaluations were conducted by experienced pathologists. MRI was consistently performed prior to revision surgery as soon as osteomyelitis was suspected. To minimize immediate postoperative changes—such as bone marrow edema, fluid collections, or soft tissue swelling—that are most prominent within the first two weeks and could interfere with diagnosing osteomyelitis, imaging was conducted at least two weeks after the most recent surgery. All MRI examinations were performed using either 1.5 Tesla (1.5 T) or 3.0 Tesla (3.0 T) scanners (Ingenia, Philips Medical Systems, Best, The Netherlands), depending on availability. The standard imaging protocol included a coronal Short Tau Inversion Recovery (STIR) sequence; an axial T1-weighted (T1w) turbo spin echo (TSE) sequence before and after administration of a gadolinium-based contrast agent with subtraction; an axial fat-suppressed T2-weighted (T2w) sequence; and a sagittal T1w fat-suppressed post-contrast sequence. Representative sequence parameters for a 1.5 T scanner are provided in Table 1.

The primary diagnostic question for both MRI and histology was the presence of osteomyelitis. The radiologists and pathologists were blinded to reduce bias. The only clinical information provided to them included lesion localization, prior trauma, and previous surgeries, but not the disease progression or additional clinical findings.

MRI findings indicative of osteomyelitis included reduced signal intensity in bone marrow on T1w sequences, focally enhanced signal intensity in bone marrow on T2w fat-suppressed sequences and STIR sequences, and focally contrast-enhanced bone marrow signal on fat-suppressed T1w sequences after administration of gadolinium-based contrast, where osteomyelitis initially presents as bone marrow edema [14,19,20]. In advanced stages of osteomyelitis, additional findings can include intraosseous abscess formation with reactive bone formation, subperiosteal abscesses, sinus tract formation, and cortical erosions.

Histological samples were formalin-fixed, paraffin-embedded, and stained with hematoxylin and eosin (H&E). Bone specimens underwent decalcification prior to processing.

### 2.4. Statistic Analysis

Characteristic features of osteomyelitis in MRI and histopathology were analyzed and compared. Descriptive statistical analysis was performed using GraphPad Prism, Version 10 (GraphPad Software, San Diego, CA, USA). The level of significance was defined at *p* < 0.05.

## 3. Results

A total of 10 patients were included in the study, with 5 assigned to the osteomyelitis (OM) group and 5 to the control group. Each group consisted of one female (20%) and four male (80%) patients. The mean age in the OM group was 59.0 ± 16.1 years, while in the control group, it was 49.8 ± 15.7 years. In both groups, three patients (60%) had sustained a monotrauma, while two patients (40%) had experienced polytrauma. Similarly, two patients (40%) in each group initially presented with an open injury, whereas three patients (60%) had a closed injury at initial presentation.

In the OM group, the affected anatomical regions were as follows: lower leg in three patients (60%), thigh in one patient (20%), and knee in one patient (20%). In the control group, the affected anatomical sites were the lower leg in three patients (60%), and the upper ankle joint in two patients (40%).

Comorbidities were evenly distributed between the groups and included cardiopulmonary diseases, vascular disorders, metabolic and endocrine diseases, as well as malignancies, as detailed in Table 2. No significant epidemiological differences were observed between the groups (*p* > 0.05).

### 3.1. Microbiology

Microbiological pathogens were detected in all OM cases within the bone (Table 3), whereas all bone samples in the control group remained sterile. In one control, no pathogen was isolated from either the bone or soft tissue.

Among the OM cases, three patients had a monomicrobial infection, two patients had a polymicrobial infection, and three patients had additional pathogens isolated from the soft tissue.

### 3.2. MRI

**Figure 1:**
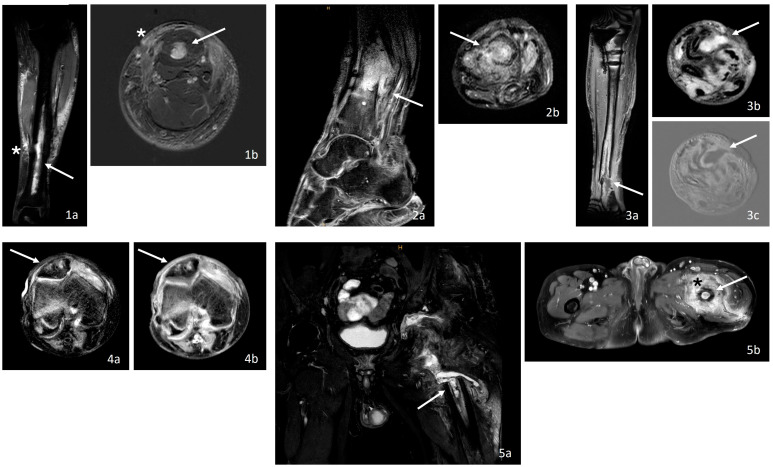
Typical osteomyelitis findings in MRI: bone marrow edema and intramedullary abscess (↑) with associated soft tissue reactions (*), also see Table 4. (**1**) MRI of the left lower leg with chronic osteomyelitis and bone marrow edema ((**a**): coronal, STIR TSE; (**b**): transverse, sT1w TSE). (**2**) MRI of the left ankle with chronic osteomyelitis including bone marrow edema and a developing intramedullary abscess ((**a**): sagittal, PDw mDIXON TSE; (**b**): transverse, T2w mDIXON TSE). (**3**) MRI of the left lower leg with acute-on-chronic osteomyelitis of the distal tibial shaft with intramedullary abscess ((**a**): coronal, T1w mDIXON TSE + contrast; (**b**): transverse, T2w mDIXON; (**c**): transverse, sT1w TSE). (**4**) MRI of the right knee with patella showing chronic osteomyelitis with intrapatellar edema and peripheral contrast enhancement as a sign of a developing intraosseous abscess ((**a**): transverse, T2w mDIXON; (**b**): transverse, T1w mDIXON TSE + contrast). (**5**) MRI of the left pelvis/hip with chronically abscessed osteomyelitis, bone marrow edema, and intramedullary abscess ((**a**): coronal, T2w TSE mDIXON; (**b**): transverse, T1w mDIXON TSE + contrast). mDIXON: modified DIXON, PDw: proton density-weighted, STIR: Short Tau Inversion Recovery, TSE: turbo spin echo, T1w: T1-weighted, T2w: T2-weighted.

In all OM cases, MRI revealed typical osteomyelitis changes (Table 4, Figure 1). Advanced findings with intraosseous abscesses were additionally present in two OM patients (40%) and early abscess formation was additionally observed in two OM cases (40%).

**Figure 2:**
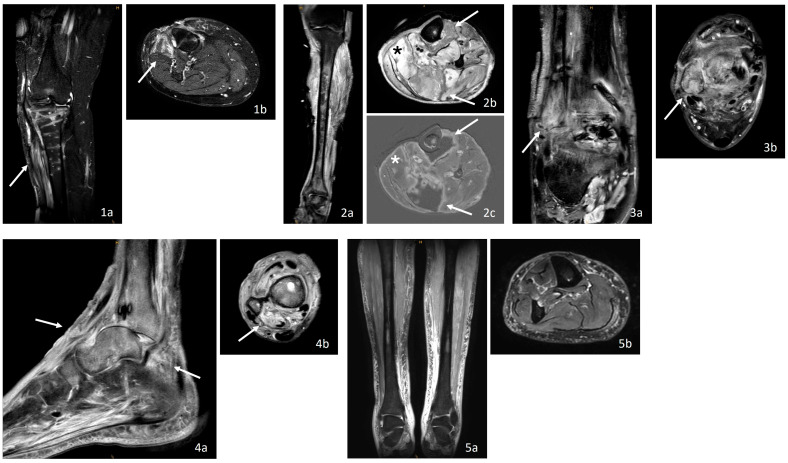
MRI findings in controls. Soft tissue edema with contrast enhancement (↑), also see Table 4. (**1**) MRI of the right lower leg with soft tissue infection without bone marrow edema ((**a**): coronal, STIR; (**b**): transverse, T2w mDIXON TSE). (**2**) MRI of the left lower leg with soft tissue infection and muscle necrosis (*) ((**a**): coronal, STIR; (**b**): transverse, T2w mDIXON TSE; (**c**): transverse, sT1w TSE). (**3**) Non-specific MRI of the right ankle with soft tissue infection and contrast enhancement in the distal fibula ((**a**): coronal, PDw mDIXON TSE MARS; (**b**): transverse, PDw mDIXON TSE MARS). (**4**) Non-specific MRI of the right ankle with soft tissue infection and subtle contrast enhancement in the adjacent bone ((**a**): sagittal, PDw mDIXON TSE; (**b**): transverse, PDw mDIXON TSE). (**5**) MRI of the right lower leg with soft tissue infection without bone marrow edema ((**a**): coronal, STIR TSE; (**b**): transverse, T2w mDIXON TSE). MARS: metal artifact reduction sequence, mDIXON: modified DIXON, PDw: proton density-weighted, STIR: Short Tau Inversion Recovery, TSE: turbo spin echo, T1w: T1-weighted, T2w: T2-weighted.

In the control group, three patients (60%) showed no osteomyelitis-specific changes. Two control patients (40%) exhibited remaining postoperative changes after previous osteosynthesis (Table 4, Figure 2), which, due to bone edema with marginal contrast enhancement, bore similarities to osteomyelitis and were therefore classified as “nonspecific”.

No subperiosteal abscesses, sinus tracts, or cortical erosions were detected in this case series; however, all cases showed evidence of soft tissue infection.

### 3.3. Histology

Histologically, all OM cases exhibited osteonecrosis with fragmented bone, a mixture of vital and non-vital bone, and evidence of bone remodeling processes. Additionally, infiltration of osteocytic lacunae by inflammatory cells, predominantly neutrophilic granulocytes, was observed. Among the OM cases, four patients (80%) also showed bone marrow fibrosis and one patient (20%) exhibited fibrin deposits.

**Figure 3:**
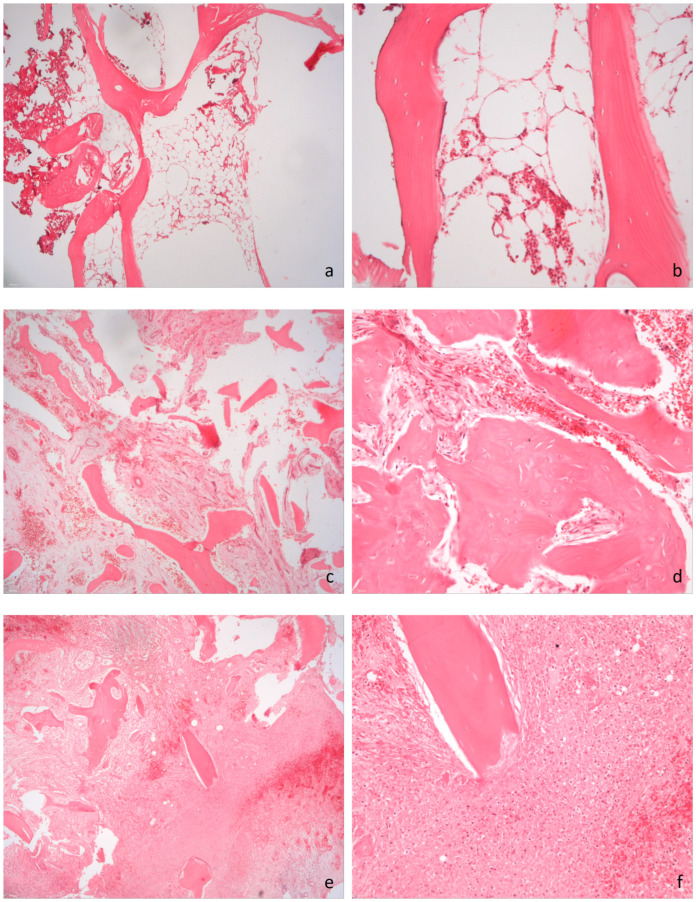
Histopathology of osteomyelitis and controls. **a**,**b**: Histological specimen of a bone sample with trabecular bone structures without signs of osteomyelitis ((**a**): magnification 5×, (**b**): magnification 20×). (**c**,**d**): Histological specimen showing chronic osteomyelitis with medullary fibrosis and diffuse granulocytic infiltration ((**c**): magnification 5×, (**d**): magnification 20×). (**e**,**f**): Histological specimen of a bone sample with trabecular bone structures and acute florid osteomyelitis with granulocytic infiltration ((**e**): magnification 5×, (**f**): magnification 20×).

In the control group, a chronic granulating inflammation of the soft tissue was present in four cases (80%), but no inflammatory infiltration was detected in any bone sample. Two control cases (40%) showed osteonecrosis without inflammatory infiltration, with one of these cases (20%) also displaying bone marrow fibrosis. The histological differences are summarized in Table 4 and illustrated in Figure 3.

## 4. Discussion

The diagnosis and treatment of osteomyelitis in the extremities remain inconsistent and non-standardized due to the lack of evidence-based guidelines. Accurate diagnosis is the cornerstone of any successful therapy. However, in early stages, diagnosis may be challenging due to ambiguous findings, potentially delaying appropriate treatment. Therefore, it is essential to utilize available diagnostic tools effectively and interpret their findings meticulously. In most cases, a combination of multiple diagnostic modalities is beneficial and may even be necessary to establish the definitive diagnosis [12]. While microbiological pathogen detection is widely regarded as the gold standard in infection diagnostics, including bone and joint infections, histological analysis of bone samples and MRI are not routinely employed or are incompletely interpreted due to a lack of awareness regarding their diagnostic value and their execution.

The aim of this study was to systematically compare osteomyelitis-specific findings in the extremities in MRI and histology using a pilot study in terms of a case-control series. By doing so, we sought to provide clinicians with characteristic diagnostic patterns and guidance on interpreting these findings, ultimately improving diagnostic accuracy and treatment decision-making.

Equally as critical as accurate interpretation of findings is ensuring proper pre-analytical procedures. This includes timely MRI diagnostics, which should ideally be performed prior to planned surgical intervention to minimize artifacts caused by surgery-related trauma [20]. To minimize these artifacts, in our study, we ensured that MRI was performed not only prior to revision surgery, but also at least two weeks after any previous osteosynthesis or bony débridement. The most definitive sign of osteomyelitis is the presence of an intramedullary abscess. Early indicators, such as bone marrow edema, are less specific, as they can also occur shortly after trauma or surgery, including fractures and operative interventions. While bone marrow edema and soft tissue swelling are most prominent within the first two postoperative weeks, they may persist—albeit with decreasing intensity—for up to 3 to 6 months [21].

Accurate interpretation of MRI findings requires knowledge of prior surgical or traumatic events. Therefore, both MRI acquisition and interpretation should preferably be conducted by radiologists with expertise in musculoskeletal imaging. In cases where findings remain ambiguous, interpretation should be integrated with clinical context and other diagnostic modalities. As highlighted in previous research, a multimodal approach—combining MRI, histology, and microbiology—is suggested and often essential, particularly in culture-negative or diagnostically inconclusive cases, to ensure an accurate diagnosis of osteomyelitis [12].

For histological bone sampling, it is advisable to obtain specimens from representative areas, ideally guided by MRI correlation whenever possible. Furthermore, preserving the integrity of bone samples is crucial, as severely traumatized specimens may no longer allow for accurate assessment of bone architecture and characteristic inflammatory cell infiltration, which are essential for diagnosing osteomyelitis. While inflammatory cell infiltration remains the hallmark of osteomyelitis, other findings such as osteonecrosis, fibrosis, and bone remodeling can also occur in non-infectious conditions. However, differentiation from malignant lesions is usually straightforward due to the absence of cellular atypia and negative immunohistochemical markers.

Typical MRI findings of osteomyelitis, including bone and bone marrow edema with contrast enhancement, were observed in all osteomyelitis cases. However, discrete bone marrow edema with contrast uptake, which may result from stress reactions or postoperative trauma, can be challenging to differentiate from early osteomyelitis, as seen in two control cases (see Figure 2). In such instances, radiological assessment and correlation with additional clinical and diagnostic findings are essential for accurate interpretation.

Although this is the first prospective study to present characteristic findings of osteomyelitis in both MRI and histology—alongside a control group without osteomyelitis—the limited sample size remains a study limitation. However, this constraint is primarily attributable to the prospective study design, the relatively low incidence of this complex disease, and the lack of standardized treatment protocols. Given the anticipated low number of cases, the study was deliberately designed as a pilot and does not aim to be confirmatory. Its main objective was to illustrate typical diagnostic phenotypes and raise awareness of possible imaging and histological patterns.

Despite the limited sample size, the study offers a valuable overview of common osteomyelitis findings and their overlap or distinction from healthy control cases. No study can fully capture the entire disease spectrum; hence, this work does not claim to be exhaustive. Instead, it serves as a practical tool to support clinical decision-making. Based on these results, a larger, confirmatory study—ideally longitudinal in design—could be conducted to validate and refine the observed patterns and assess long-term outcomes using comprehensive diagnostic methods. In a time when healthcare systems face increasing financial pressures, precise and timely diagnostics are essential for guiding effective treatment pathways. Accurate diagnosis not only helps avoid unnecessary, repeated, or prolonged interventions and hospital stays—benefiting both healthcare providers and patients—but also supports more efficient use of limited resources. While not all facilities may be equipped to provide the full spectrum of treatment options, awareness of this complex condition and its diagnostic hallmarks remains critical. Recognizing osteomyelitis early allows for appropriate initial management or, if needed, timely referral to specialized centers with multidisciplinary expertise. Ultimately, the clinician’s ability to initiate the correct treatment pathway can make the difference between successful limb salvage and the need for amputation.

In the present study cohort, a microbiological pathogen was successfully identified in all OM cases, serving as an inclusion criterion. However, since microbiological confirmation is not always feasible, particularly in chronic cases [17], additional diagnostic modalities, such as histology and MRI, are essential for accurate diagnosis. In cases of persistent uncertainty or incongruent findings, reevaluation of preanalytics or even repeat diagnostics should be strongly considered.

## 5. Conclusions

Bacterial osteomyelitis after trauma remains a severe complication, predominantly affecting the lower extremities. A thorough understanding of the available diagnostic modalities is essential for accurate and effective treatment. In addition to microbiological analysis, MRI and histopathology are currently the diagnostic methods of choice. This case-control pilot study is the first to systematically present and differentiate characteristic findings of post-traumatic bacterial osteomyelitis in the lower extremities in both histology and MRI from those observed in controls. These findings may aid clinicians in the assessment and interpretation of diagnostic results within the broader context of osteomyelitis, thereby supporting therapeutic decision-making while also improving cost-effectiveness and patient outcomes.

## Figures and Tables

**Table 1:** MRI protocol and sequence parameters at 1.5 Tesla for identification of osteomyelitis in the lower limbs.

Parameter	Pre-/Post-Contrast T1w TSE Axial	T2x mDIXON TSE Axial	STIR TSE Coronal	Post-Contrast T1w SPIR TSE Sagittal
TR/TE (ms)	687/8	4120/80	4596/65	615/10
Flip angle (degrees)	90	90	90	90
Field of view (mm)	380 × 380	380 × 380	480 × 480	460 × 460
Matrix	377 × 508	431 × 544	248 × 368	563 × 768
Reconstructed Voxel size (mm)	0.59 × 0.59 × 6.00	0.44 × 0.44 × 6.00	0.71 × 0.71 × 4.00	0.40 × 0.40 × 3.0
Slices	74	74	36	30
TSE factor	6	18	24	5
Acquisition time (min:s)	3:39.9	5:13.1	2:08.7	3:46.3
PI reduction factor (SENSE)	1.7	2.0	1.0	1.0
NSA	1	1	1	1

min:s minutes/seconds, NSA: number of signal averages, PI parallel imaging, SPIR: spectral presaturation with inversion recovery, STIR: Short Tau Inversion Recovery, TE: time to echo, TR: time of repetition, TSE: turbo spin echo, T1w: T1-weighted, T2w mDIXON: T2-weighted modified DIXON.

**Table 2:** Comorbidities.

Comorbidities		OM Group	Control Group
Cardiovascular disorders	Arterial hypertension	n = 2	n = 1
	Cardiac arrhythmia	n = 1	n = 1
	Coronary heart disease	n = 0	n = 1
Vascular disorders	PAD ^1^	n = 1	n = 1
	Chronic venous insufficiency	n = 1	n = 0
Pulmonary disorders	COPD ^2^	n = 1	n = 0
Metabolic/endocrinological disorders	Type 2 diabetes mellitus	n = 1	n = 0
	Type 1 diabetes mellitus	n = 0	n = 1
Autoimmune disorders	Psoriasis	n = 0	n = 1
Malignant tumors	Pancreatic/ovarian cancer	n = 0	n = 2
	Lymphadenopathy	n = 1	n = 0
Others	Chronic renal insufficiency	n = 1	n = 0
	Hypercholesterolemia	n = 0	n = 1
	Neurological/psychiatric illness	n = 1	n = 1
	Alcohol abuse	n = 0	n = 1
	Nicotine abuse	n = 0	n = 1
	Drug addiction	n = 1	n = 1

^1^ PAD: Peripheral artery disease, ^2^ COPD: Chronic obstructive pulmonary disease.

**Table 3:** Microbiologic pathogens of osteomyelitis.

OM Group	Pathogen in Bone	Other Pathogens
1	*S. haemolyticus*	*Escherichia coli*
		*S. aureus* (MSSA *)
2	*S. epidermidis*	-
	*Pseudomonas aeruginosa*	
3	*S. aureus* (MSSA *)	*S. epidermidis*
		*Bacillus cereus*
4	*S. epidermidis*	-
5	*S. aureus* (MSSA *)	*S. epidermidis*
	*S. saccharolyticus*	*S. pettenkoferi*

* MSSA: Methicillin-susceptible *Staphylococcus aureus*.

**Table 4:** MRI and histopathologic findings of osteomyelitis.

Pat., Fig. No.	Affected Localization (OM, Fx)	MRI Findings of the Bone	Interpretation	Histopathologic Findings	Interpretation
Osteomyelitis group					
1, Figure 1.1	OM: Left lower leg, Fx: Lower leg shaft fracture	Long-distance circumscribed CM enhancement of the bone marrow in the tibial shaft (↑) and the periosteal soft tissue with edema (*)	Characteristic of OM	Spongy bone components with fragmented trabeculae and mildly increased bone remodeling, edema in the marrow spaces, as well as fibrosis and lymphoid cell accumulations	Chronic granulating OM
2, Figure 1.2	OM: Left ankle, Fx: Lower leg shaft fracture	CM enhancement of the bone marrow adjacent to the fracture zone (↑) in the sense of bone edema and intraosseous abscess with marked soft tissue reaction	Characteristic of OM	Vital, focally avital corticospongious bone with signs of remodeling, reactive trabecular bone formation, and marrow fibrosis with acute and chronically granulating inflammation in the soft tissue and marrow	Active and chronic granulating OM
3, Figure 1.3	OM: Left lower leg, Fx: Proximal tibia and lower leg shaft fracture	Intramedullary fluid accumulation with CM enhancement as a sign of an intramedullary abscess of the distal tibia (↑)	Characteristic of OM	Acute and chronically granulating inflammation in the periosteal soft tissue and marrow, partially vital, partially avital bone with remodeling processes, marrow fibrosis, and dense infiltrates of neutrophilic granulocytes, lymphocytes, and plasma cells, along with areas of tissue necrosis and fibrin deposits	Active and chronic granulating OM
4, Figure 1.4	OM: Right knee and patella, Fx: Patella fracture	Intrapatellar inhomogeneous CM enhancement as a sign of bone edema with peripheral enhancement (↑) in the context of an intraosseous abscess	Characteristic of OM	Acute purulent inflammation of the soft tissue and bone with loose medullary fibrosis, necrobiotic spongy lamellar bone with signs of remodeling, and infiltration of neutrophilic granulocytes	Chronic granulating OM
5, Figure 1.5	OM: Left pelvis and hip, Fx: Proximal femur fracture	Pronounced bone marrow edema with intramedullary abscess (↑) and soft tissue reaction coronary as well as long-distance CM enhancement intramedullary (↑) and in the periosteal tissue (*) axial	Characteristic of OM	Chronic granulomatous inflammation in bone and soft tissue with fragmented bone tissue undergoing remodeling processes	Chronic granulating OM
Control group					
6, Figure 2.1	Right lower leg, Fx: Proximal tibia fracture	Low-grade intramedullary edema without BM accumulation in the context of postoperative changes in periosteal soft tissue reaction with edema and BM accumulation (↑) as a sign of soft tissue infection, but no signs of intramedullary sequestration or abscesses	Unremarkable	Periprosthetic wear-type membrane in the soft tissue, with no evidence of inflammatory cells in the bone	Soft tissue infection
7, Figure 2.2	Left lower leg, Fx: Ankle and proximal fibula fracture	Pronounced edematous distension of the lower leg muscles (*) with partial muscle necrosis (↑) (axial differences in sT1w and T2w), slight bone marrow edema of the tibial shaft, but no relevant CM enhancement, no bone necrosis or inflammatory changes	Unremarkable	Chronically granulating soft tissue inflammation with partially non-vital, atrophic skeletal muscle and focal acute granulocytic inflammation, without evidence of inflammatory cells in the bone	Soft tissue infection
8, Figure 2.3	Right ankle, Fx: Ankle fracture	Minimal CM enhancement of the superficial cortex and discrete bone marrow edema with CM enhancement of the distal fibula (↑) and adjacent soft tissues, primarily reactive postoperatively	Non-specific	Partially vital and non-vital, fragmented, inflammation-free bone tissue with remodeling processes, at most focal cell residues within otherwise empty osteocytic lacunae, interspersed with fibrin strands, gangrene of skin/subcutaneous tissue, and chronic granulomatous and fibrosing soft tissue inflammation	Soft tissue infection, osteonecrosis
9, Figure 2.4	Right ankle, Fx: Talus and distal fibula fracture	Soft tissue edema (↑) and focal bone edema with diffuse contrast enhancement, but no signs of cortical destruction, abscesses, or sequestra in the context of a subluxated ankle joint, most likely indicative of a stress reaction	Non-specific	Bone tissue with medullary fibrosis but without inflammatory infiltration	Osteonecrosis
10, Figure 2.5	Right lower leg, Fx: Tibia shaft fracture	Soft tissue reaction without osseous involvement in terms of bone edema, no contrast enhancement, no sequestra, abscesses, or cortical reactions	Unremarkable	Focal purulent inflammatory reaction in the soft tissue without a corresponding inflammatory response in the bone	Soft tissue infection

CM: contrast medium, Fx: fracture, OM: osteomyelitis. Figure 1 and Figure 2 present the corresponding entities, which are indicated by the symbols (↑) and (*).

## Data Availability

The original contributions presented in this study are included in the article. Further inquiries can be directed to the corresponding author(s).

## Abbreviations

The following abbreviations are used in this manuscript:

CM	Contrast medium
COPD	Chronic obstructive pulmonary disease
Fx	Fracture
MARS	Metal artifact reduction sequence
mDIXON	Modified DIXON
MSSA	Methicillin-susceptible Staphylococcus aureus
NSA	Number of signal averages
OM	Osteomyelitis
PAD	Peripheral artery disease
PI	Parallel imaging
PDw	Proton density-weighted
SPIR	Spectral presaturation with inversion recovery
STIR	Short Tau Inversion Recovery
TSE	Turbo spin echo
T1w	T1-weighted
T2w	T2-weighted
